# Tranexamic Acid for Shoulder Arthroplasty: A Systematic Review and Meta-Analysis

**DOI:** 10.3390/jcm11010048

**Published:** 2021-12-23

**Authors:** Jaroslaw Pecold, Mahdi Al-Jeabory, Maciej Krupowies, Ewa Manka, Adam Smereka, Jerzy Robert Ladny, Lukasz Szarpak

**Affiliations:** 1 Department of Trauma and Orthopedic Surgery, Ruda Slaska City Hospital, 41-703 Ruda Slaska, Poland; Jarekpecold@Tlen.pl (J.P.); mmahdi@interia.pl (M.A.-J.); 2 Research Unit, Polish Society of Disaster Medicine, 05-806 Warsaw, Poland; krupowiesmaciej@gmail.com (M.K.); Jerzy.r.ladny@gmail.com (J.R.L.); 3 Department of Internal Medicine, Angiology and Physical Medicine in Bytom, Faculty of Medical Sciences in Zabrze, Medical University of Silesia in Katowice, 41-800 Zabrze, Poland; ewa.irena.manka@gmail.com; 4 Department of Gastroenterology and Hepatology, Faculty of Medicine, Wroclaw Medical University, 53-126 Wroclaw, Poland; adam.smereka@umw.edu.pl; 5 Department of Emergency Medicine, Bialystok Medical University, 15-026 Bialystok, Poland; 6 Institute of Outcomes Research, Maria Sklodowska-Curie Medical Academy, 03-411 Warsaw, Poland; 7 Research Unit, Maria Sklodowska-Curie Bialystok Oncology Center, 15-027 Bialystok, Poland

**Keywords:** tranexamic acid, TXA, shoulder, arthroscopy, bleeding, systematic review, meta-analysis

## Abstract

Tranexamic acid (TXA) is an antifibrinolytic agent that has been shown to decrease blood loss and transfusion rates after knee and hip arthroplasty, however with only limited evidence to support its use in shoulder arthroplasty. Therefore, we performed a systematic review and meta-analysis to evaluate the clinical usefulness of tranexamic acid for shoulder arthroplasty. A thorough literature search was conducted across four electronic databases (PubMed, Cochrane Library, Web of Science, Scopus) from inception through to 1 December 2021. The mean difference (MD), odds ratio (OR) or relative risk (RR) and 95% confidence interval (CI) were used to estimate pooled results from studies. Total of 10 studies comprising of 993 patients met the inclusion criteria and were included in the analysis. Blood volume loss in the TXA and non-TXA group was 0.66 ± 0.52 vs. 0.834 ± 0.592 L (MD= −0.15; 95%CI: −0.23 to −0.07; *p* < 0.001). Change of hemoglobin levels were 2.2 ± 1.0 for TXA group compared to 2.7 ± 1.1 for non-TXA group (MD= −0.51; 95%CI: −0.57 to −0.44; *p* < 0.001) and hematocrit change was 6.1 ± 2.7% vs. 7.9 ± 3.1%, respectively; (MD= −1.43; 95%CI: −2.27 to −0.59; *p* < 0.001). Tranexamic acid use for shoulder arthroplasty reduces blood volume loss during and after surgery and reduces drain output and hematocrit change.

## 1. Introduction

Tranexamic acid (TXA) is an antifibrinolytic that inhibits fibrin’s plasmin-mediated degradation and is used to stabilize clots and reduce active bleeding. In orthopaedic surgery, tranexamic acid is most notably involved in the elective orthopaedic procedures necessitating transfusion [1,2]. The use of tranexamic acid has become widely accepted in total knee and hip arthroplasty to prevent extensive blood loss and lower transfusion rates, but it can be also beneficial for patients who undergo total shoulder arthroplasty [3]. A significant benefit of TXA in several types of orthopaedic surgery may also be a reduction in the need for blood product transfusions, reduced hospital costs, laboratory costs and shorter hospital stays [3]. When considering the benefits of TXA, it is also essential to consider the risk for increased thromboembolic events and provide post-operative thromboprophylaxis [4].

Shoulder-scapular-joint alloplasty procedures have become increasingly popular in recent years. Modern implants provide various surgical options, depending on the indications and anatomical conditions. Although the number of possible complications is still high, the results of revision surgery are improving [5]. Shoulder alloplasty can be divided into the partial and total shoulder [5]. Total shoulder arthroplasty is divided into anatomic total shoulder arthroplasty (ATSA) and reverse total shoulder arthroplasty (RTSA) [6]. One method used to improve the procedure’s effectiveness and reduce possible bleeding complications and the need for blood transfusions is the perioperative use of tranexamic acid [7]. Importantly, perioperative use of TXA does not appear to significantly increase the risk of incident embolic and thrombotic events [8], including patients with a history of similar incidents [9]. Additionally, perioperative use of tranexamic acid at a dose of 20 mg/kg body weight shortens the recovery time of patients and contributes to shorter hospitalizations [10].

We already know that TXA reduces blood loss in shoulder arthroplasty, but the benefits and risks of using tranexamic acid are still unclear. Meta-analysis of data from different studies may facilitate clinical decision-making regarding the use of TXA, including support for accurate assessment of some rare complications and their clinical significance.

Therefore, we performed a systematic review and meta-analysis to evaluate the clinical usefulness of tranexamic acid of the shoulder arthroplasty.

## 2. Materials and Methods

### 2.1. Search Strategy

The study was designed as a systematic review and meta-analysis and was performed in accordance with the Preferred Reporting Items for Systematic Reviews and Meta-Analysis (PRISMA) statement [11].

In this systematic review and meta-analysis, we searched PubMed, Cochrane Library, Web of Science, Scopus from the databases’ inception to 1 December 2021 for original, peer-reviewed primary research articles, including observational or interventional studies, that describe the clinical usefulness of tranexamic acid for shoulder arthroplasty. We searched the following terms: “tranexamic acid” OR “TXA” AND “shoulder”. Additionally, the reference lists of retrieved articles were also reviewed to identify additional eligible studies. To avoid double data counting, when there were multiple publications from the same trial sample, the one with the largest sample size was included.

### 2.2. Inclusion Criteria

Studies that were included in this meta-analysis had to fulfil the following PICOS criteria: (1) Participants, patients with 18 years old or older requiring shoulder arthroplasty; (2) Intervention, tranexamic acid treatment; (3) Comparison, non-TXA treatment; (4) Outcomes, operative data and adverse events occurrence; (5) Study design, randomized controlled trials and retrospective trials comparing TXA and non-TXA care for their effects in patients with shoulder arthroplasty. Studies were excluded if they were reviews, animal studies, case reports, letters, conference or poster abstracts, or articles not containing original data.

### 2.3. Data Extraction

Data extraction was performed by two reviewers (J.P. and M.A.-J.). From the studies that met the inclusion eligibility criteria, the following data were extracted into a predefined Microsoft Excel spreadsheet (Microsoft Corp., Redmond, WA, USA): (a) study characteristic (i.e., first author, year of publication, country, study design, inclusion and exclusion criteria, primary outcomes, findings; intervention and control group); (b) participant characteristics (i.e., number of participants, age, sex); (c) main study outcomes (i.e., blood volume loss, operative time, adverse events, hospital length of stay). Potential disagreements were resolved by discussion with a third reviewer (L.S.).

### 2.4. Quality Assessment

Two reviewers (J.P. and M.A.-J.) evaluated the quality of each study. Potential disagreements were discussed and resolved in a consensus meeting with the third reviewer (L.S.). The RoB 2 tool (revised tool for risk of bias in randomized trials) was used to assess the quality of randomized studies [12], and the ROBINS-I tool (tool to determine the risk of bias in non-randomized studies of interventions) was used to assess the quality of non-randomized trials [13]. The risk of bias assessments was visualized using the Robvis application [14].

### 2.5. Statistical Analysis

The meta-analysis was conducted using the Review Manager, version 5.4EN (RevMan; The Cochrane Collaboration, Oxford, UK). The significance level was set at 0.05 for all analyses. When the continuous outcomes were reported in a study as median, range, and interquartile range, we estimated means and standard deviations using the formula described by Hozo et al. [15]. The mean difference (MD), odds ratio (OR) or relative risk (RR) and 95% confidence interval (CI) were used to estimate pooled results from studies. The Q and I^2^ statistic were used to investigate heterogeneity among the studies. A fixed model effect was used when I^2^ < 50%, and a random model effect was used in other cases.

## 3. Results

### 3.1. Eligible Studies and Study Characteristics

**Figure 1:**
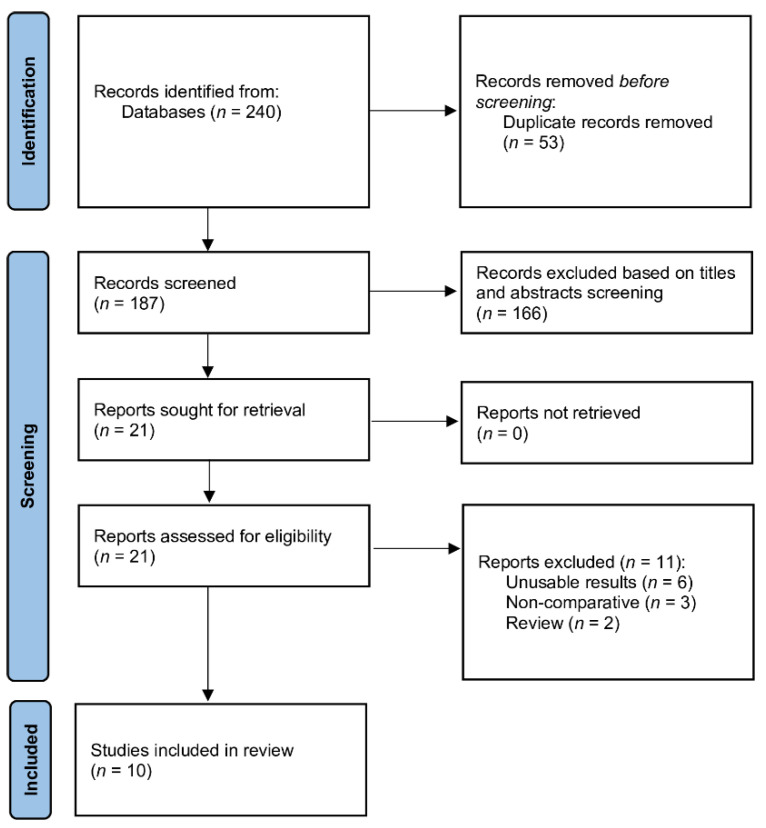
Forest plot of blood volume loss among TXA and non-TXA groups. The centre of each square represents the weighted mean differences for individual trials, and the corresponding horizontal line stands for a 95% confidence interval. The diamonds represent pooled results.

The database search identified 240 citations (Figure 1). After excluding duplicates, reviews, editorials, letters, case reports and meta-analysis, a total of ten studies [10,16,17,18,19,20,21,22,23,24] met the inclusion and exclusion criteria and were included in the analysis, comprising 993 patients.

The mean age of participants in TXA and non-TXA groups was 59.6 ± 21.5 and 60.3 ± 21.3 years, respectively. The men constituted 51.5% vs 49.4%, respectively, for TXA and non-TXA groups. Baseline characteristics for all included studies are shown in Table 1 and Table S1. Studies were published from 2015 to 2021. Seven studies were designed as randomized controlled trials [17,18,19,20,21,23,24]. The results of the assessment of risk of bias among included studies are provided in Figures S1–S4.

### 3.2. Meta-Analysis

**Figure 2:**
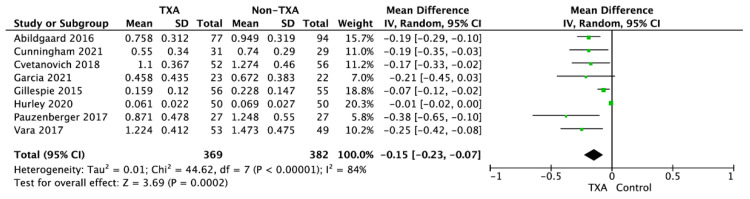
Forest plot of blood volume loss among TXA and non-TXA groups. The centre of each square represents the weighted mean differences for individual trials, and the corresponding horizontal line stands for a 95% confidence interval. The diamonds represent pooled results. Legend: CI = confidence interval; SD = standard deviation.

Blood volume loss in the TXA and non-TXA group varied and amounted to 0.66 ± 0.52 vs. 0.834 ± 0.592 L (MD = −0.15; 95%CI: −0.23 to −0.07; I^2^ = 84%; *p* < 0.001; Figure 2).

Drain output was reported in six studies. Polled analysis of drain output was 110.5 ± 100.4 mL for TXA group, and 222.9 ± 187.2 mL for non-TXA group (MD = −92.51; 95%CI: −141.09 to −43.93; I^2^ = 92%; *p* < 0.001).

Change of hemoglobin levels form preoperatively to postoperatively periods were reported in six trials and were 2.2 ± 1.0 for TXA group compared to 2.7 ± 1.1 for non-TXA group (MD = −0.51; 95%CI: −0.57 to −0.44; I^2^ = 0%; *p* < 0.001).

Hematocrit change was reported in five studies and was statistically smaller in TXA group (6.1 ± 2.7%), compared to non-TXA group (7.9 ± 3.1%); MD= −1.43; 95%CI: −2.27 to −0.59; I^2^ = 95%; *p* < 0.001).

**Figure 3:**
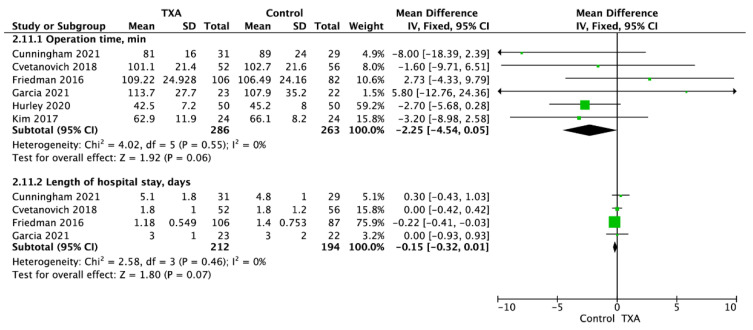
Forest plot of (**2.11.1**) operation time; (**2.11.2**) length of hospital stay among TXA and non-TXA groups. The centre of each square represents the weighted mean differences for individual trials, and the corresponding horizontal line stands for a 95% confidence interval. The diamonds represent pooled results.

Operation time was reported in six trials and was 89.5 ± 33.0 min for TXA group compared to 88.5 ± 32.2 min for non-TXA group (MD = −2.25; 95%CI: −4.54 to 0.05; I^2^ = 0%; *p* = 0.06; Figure 3). Length of hospital stay in TXA and non-TXA (control) groups was 2.1 ± 1.7 vs. 2.2 ± 1.6 days, respectively (MD = −0.15; 95%CI: −0.32 to 0.01; I^2^ = 0%; *p* = 0.07).

Polled analysis of three studies showed that 3.0% of patients in the TXA group and 3.5% in the non-TXA group required transfusion (RR = 0.56; 95%CI: 0.20 to 1.59; *p* = 0.28). Revision was no required in TXA compared to 0.9% of cases in non-TXA group (RR = 0.33; 95%CI: 0.01 to 7.99; *p* = 0.50). Hematoma was observed in 20.4% in the TXA group and 53.8% in non-TXA group (RR = 0.39; 95%CI: 0.27 to 0.57; *p* < 0.001). Thromboembolic complications were not noted in any of the groups.

## 4. Discussion

TXA is currently a commonly used perioperative antifibrinolytic agent. The antifibrinolytic is used in surgery but also in various other bleeding manifestations in congenital coagulopathies, such as fibrinogen deficiency [25]. With the increase in the number of TSAs performed, and the constant expansion of the indications qualifying for the procedure, there is a need to reduce postoperative complications and improve the procedure’s results.

In this study, we evaluate results from three retrospective studies [10,16,22] and seven RCTs that compared outcomes in the TXA group and non-TXA group [17,18,19,20,21,23,24]. The main findings of this meta-analysis relate to nine factors, which we have sorted out for clarity: blood volume loss, hematocrit change, length of hospitalization, operation time, hematoma, drain output, need for revision and thromboembolic complications. In a retrospective study including an analysis of 155 complications after TSA, Anthony et al. highlights that the most common complication is bleeding-requiring transfusion [26]. Our study identified a notably increased blood volume loss in the non-TXA group of 0.834 ± 0.592 L compared to 0.66 ± 0.52 L in the TXA group, which is also reflected in the change in hematocrit values, whose change for the described groups was 7.9 ± 3.1% for the non-TXA group compared to TXA group 6.1 ± 2.7%, respectively. TXA successfully prevents perioperative blood loss. This decreases postoperative pain and reduces complications, postoperative mortality, and the length of hospitalization. At the same time, TXA has side effects limited to nausea and diarrhoea, making it a well-tolerated drug [27]. These benefits also carry a reduction in costs associated with postoperative care. As reported by Kandil et al. in 2016, the hospitalization time for patients after TSA who required a blood transfusion is 1.8 days longer which is $11,794 more, compared to patients who did not receive a transfusion [28]. In our meta-analysis results, the hospitalization time of patients in the TXA group is 2.1 ± 1.7 vs. 2.2 ± 1.6 in the non-TXA group. This emphasizes the need for further in-depth analyses of the available studies due to the discrepancy with the reports mentioned above of Kandil et al., especially considering the difference we also showed in the duration of the operation itself. These times differed slightly (TXA 89.5 ± 33.0 min vs non-TXA 89.1 ± 32.2 min) but in the study by Friedman et al. performed on a group of 194 patients, the time in the recovery room in the TXA group was shorter on average by as much as 24 min [10].

Wang et al. in a 2021 study showed that patients with moderate to severe preoperative anemia were at increased risk of cardiac and pulmonary complications, postoperative blood transfusion, prolonged length of stay, reoperation, and death [29]. In our assessment, these results are transferable to the postoperative situation because it has been proven that TSA without antifibrinolytics is associated with high blood loss. According to studies, the volume of blood lost in the first postoperative day ranges from 159 to 1473 mL [10,16,17,18,19,20,21,23,24].

The reduction in postoperative pain is also associated with a significant difference in the incidence of postoperative hematoma. In our meta-analysis, the occurrence rate in the TXA group was 20.4%, while hematoma formation in non-TXA patients occurred 53.8%. Hematomas which result from continuous blood loss cause painful swelling, which contributes to the use of opioid medications and may even result in the need for surgical drainage, which directly delays hospital discharge time [17,21]. Moreover, persistent postoperative drainage may increase the risk of tissue contamination and deep infection, which prolongs the time and cumulates the costs of treatment [30,31].

Among the studies we analyzed that used drain output, only Cvetanovich et al. reported no statistically significant difference in drainage volume [18]. The other studies show a reduced volume in the TXA group (109.9 ± 104.3 mL) versus the non-TXA group (254.4 ± 200.5 mL). This difference shows the need for additional large randomized controlled trials. Additionally, in the two studies we analyzed, the TSA requiring revision surgery percentage was 0.9% in the non-TXA group, whereas patients after TXA application did not require revision. This may be related to the reported lower complications resulting from less blood loss in patients who received an antifibrinolytic agent.

Our meta-analysis also highlighted the finding of Cvetanovich et al. [18]. As indicated in the study, thromboembolic complications were not noted in any of the groups. This aspect is particularly relevant given that deep vein thrombosis and pulmonary embolism are considered the leading complications of orthopedic surgery [31,32]. This is consistent with other meta-analyses and RCTs that evaluated the safety of TXA in total joint arthroplasty. No increase in the risk of thromboembolic complications was demonstrated.

It is worth pointing out that in five analyzed studies, TXA was administered intravenously, only Gillespie et al. used in the treatment group 100 mL of normal saline infused with 2 g of TXA poured into the surgical wound and left in place for 5 min. To the best of our knowledge, there have not been many studies comparing these two methods of TXA application. Budge et al. in 2019 conducted a retrospective review on a group of only three patients comparing whether Intravenous and topical TXA are equivalent in improving postoperative hemoglobin in TSA [33]. However, Li et al., in a meta-analysis, evaluated the efficiency and safety of combined use of intravenous and topical versus single intravenous TXA [34]. The meta-analysis concerns total primary knee and hip arthroplasty. The study team shows a statistically significant reduction in total blood loss with the combined application with no increase in the risk of thromboembolic complications [35]. The findings from our statistics and the described studies demonstrate the need to compare TXA application methods further.

In performed statistical analysis, we reported significant benefits for TXA in all described aspects. However, significant heterogeneity between the compared studies was demonstrated in terms of total blood loss or drain output. This phenomenon cannot be sufficiently explained by possible differences in the anaesthetic protocol used, the thromboembolic prophylaxis plan or the way TXA was applied. In the opinion of our research team, these differences may be due to individual variability of patients, inclusion or exclusion criteria, differences in surgical protocols and different methods of measuring outcomes.

This study has limitations related to the relatively small number of studies included and to its retrospective nature. However, the data collected indicate statistically and clinically significant findings regarding the manner and safety of TXA application. Because of the limited high-quality evidence currently available, there is a need for further in-depth analysis of the available studies in terms of the most beneficial way of TXA application.

## 5. Conclusions

Tranexamic acid use for shoulder arthroplasty reduces blood volume loss during and after surgery and reduces drain output and hematocrit change.

## Figures and Tables

**Table 1:** Study characteristics.

Study	Country	Study Design	TXA Group				Non-TXA Group		
			No	Age	Sex, Male	TXA Dose	No	Age	Sex, Male
Abildgaard et al. 2016	USA	Retrospective	77	71.6 ± 10.2	49 (63.6%)	1 g	94	72.9 ± 9.4	51 (54.3%)
Cunningham et al. 2021	Switzerland	RCT	31	72 ± 8	11 (35.5%)	2 g	29	73 ± 9	6 (20.7%)
Cvetanovich et al. 2018	USA	RCT	52	67.7 ± 10.9	23 (44.2%)	1 g	56	65.2 ± 9.2	28 (50.0%)
Friedman et al. 2016	USA	Retrospective	106	NS	46 (43.4%)	20 mg/kg	88	NS	33 (37.5%)
Garcia et al. 2021	Portugal	RCT	23	76.7 ± 7.1	4 (17.4%)	1 g	22	75.7 ± 5.7	3 (13.6%)
Gillespie et al. 2015	USA	RCT	61	67.59	25 (40.9%)	2 g	57	66.45	27 (47.4%)
Hurley et al. 2020	Ireland	RCT	50	25.1 ± 6.5	48 (96.0%)	1 g	50	23.8 ± 3.4	48 (96.0%)
Kim et al. 2017	Republic of Korea	Retrospective	24	73.2 ± 4.4	3 (12.5%)	0.5 g	24	74.2 ± 4.4	6 (25.0%)
Pauzenberger et al. 2017	Austria	RCT	27	70.3 ± 9.3	20 (74.1%)	1 g	27	71.3 ± 7.9	18 (66.7%)
Vara et al. 2017	USA	RCT	53	67 ± 9	20 (37.7%)	20 mg/kg	49	66 ± 9	22 (44.9%)

Legend: NS = not specified; RCT = randomized controlled trial; TXA = tranexamic acid.

## Data Availability

Not applicable.

## Supplementary Materials

The following supporting information can be downloaded at: https://www.mdpi.com/article/10.3390/jcm11010048/s1, Table S1: Methodology characteristics among included trials, Figure S1: A summary table of review authors’ judgements for each risk of bias item for each randomized study, Figure S2: A plot of the distribution of review authors’ judgements across randomized studies for each risk of bias item, Figure S3: A summary table of review authors’ judgements for each risk of bias item for each non-randomized study, Figure S4: A plot of the distribution of review authors’ judgements across non-randomized studies for each risk of bias item.
