# Supplementary material for: Tranexamic Acid for Shoulder Arthroplasty: A Systematic Review and Meta-Analysis
Source: J Clin Med. 2021 Dec 23;11(1):48. doi: 10.3390/jcm11010048 (PMC8745038; doi:10.3390/jcm11010048)
Supplement: Supplementary file 1 [file jcm-11-00048-s001.zip › jcm-1523525-supplementary.pdf]

TRANEXAMIC ACID FOR SHOULDER ARTHROPLASTY:

A SYSTEMATIC REVIEW AND META-ANALYSIS

<u>Supplementary Digital File</u>

CONTENT:

Table S1. Methodology characteristics among included trials. 2

Figure S1. A summary table of review authors' judgements for each risk of bias item for each randomized study. 6

Figure S2. A plot of the distribution of review authors' judgements across randomized studies for each risk of bias item. 6

Figure S3. A summary table of review authors' judgements for each risk of bias item for each non-randomized study. 7

Figure S4. A plot of the distribution of review authors' judgements across non-randomized studies for each risk of bias item. 7

Table S1. Methodology characteristics among included trials.

Study	Inclusion criteria	Exclusion criteria	Primary outcome(s)	Findings
Abildgaard et al. 2016	Patients undergoing total shoulder arthroplasty and reverse total shoulder arthroplasty.	Patients younger than 50 years (5 shoulders), procedures related to an oncologic diagnosis (1 shoulder), and patients with incomplete preoperative or postoperative laboratory data (2 shoulders).	Perioperative blood loss and drain output.	Use of TXA perioperatively among patients undergoing primary shoulder arthroplasty can decrease perioperative blood loss, change in Hgb and Hct, and postoperative drain output.
Cunningham et al. 2021	Any patient undergoing primary anatomic or reverse shoulder replacement who consented to participate in the study.	Allergy to TXA, history of seizure, revision arthroplasty, refusal to undergo potential blood transfusion, and any known coagulopathy or preoperative use of anticoagulant agents.	Preoperative hemoglobin (Hb) and hematocrit levels.	A single dose of 2 g of intravenous TXA decreases blood loss and drain tube output in primary anatomic and reverse arthroplasty of the shoulder. No differences were detected in the occurrence of complications, need for transfusion, pain score, or length of hospital stay. With the mounting evidence now available, patients undergoing elective primary shoulder arthroplasty should be given intravenous TXA to decrease perioperative blood loss.
Cvetanovich et al. 2018	Patients undergoing a unilateral primary anatomic or reverse total shoulder arthroplasty.	Allergy to TXA, acquired disturbances of color vision, preoperative use of anticoagulant therapy within 5 days of surgery, history of arterial or venous thromboembolic disease (including deep venous thrombosis, pulmonary embolism, stroke, transient ischemic attack), ongoing pregnancy or breast-feeding, recent myocardial infarction	Postoperative blood loss based on a formula accounting for initial patient hemoglobin, the lowest postoperative hemoglobin, and patient blood volume approximated based on patient sex, height, and weight.	Intravenous TXA reduced blood loss after primary total shoulder arthroplasty compared with placebo.

		(within 6 months before surgery), cardiac stent placement, renal impairment, hemophilia, refusal of blood products, revision TSA, TSA performed for the indications of acute proximal humeral fracture, or prior open shoulder surgery, including failed.		
Friedman et al. 2016	Patients undergoing primary total shoulder arthroplasty.	Patients required revision.	The effects of intravenous TXA on blood loss and patient outcomes after total shoulder arthroplasty.	TXA 20 mg/kg intravenously given just before primary anatomic and reverse TSA results in statistically significant reductions in blood loss. Patients spent 21% less time in the recovery room and had a 16% shorter hospitalization, resulting in financial savings for the hospital.
Garcia et al. 2021	Patients with more than 18 years of age and with the following indications for surgery: cuff tear arthropathy, proximal humeral fractures, chronic instability, primary osteoarthrosis, and failures of previous prosthesis.	Patients with known allergy to TXA, thromboembolic event in the previous year and refusal to be transfused, to perform regional anesthesia or give written informed consent	Blood loss.	TXA use significantly decreased blood loss measured by drain output and Hb drop in TSA under regional anesthesia.
Gillespie et al. 2015	Participants underwent primary TSA or primary reverse total shoulder arthroplasty.	Revision surgery, history of cardiac disease, liver disease, renal disease, preoperative hemoglobin level <11.5 g/dL or hematocrit <35%, severe joint deformity, history of joint infection, history of bleeding or metabolic disorder, history of peripheral vascular disease,	Postoperative blood loss.	In this cohort of patients, those treated with TXA experienced a significantly lower amount of postoperative blood loss and a significantly smaller change in hemoglobin level compared with those treated with placebo. Further work is required to determine the effectiveness and clinical significance of TXA in reducing transfusion requirements in shoulder arthroplasty and,

		history of prior deep venous thrombosis (DVT) or pulmonary embolism (PE), any patient un- willing to accept a blood transfusion, and any patient with a documented allergy to TXA		more specifically, shoulder arthro- plasty performed for complicated patients or for trauma and fracture patients.
Hurley et al. 2020	All patients scheduled to undergo the Latarjet procedure for anterior shoulder instability	Refusal to participate in the study, revision shoulder stabilization, known allergy to TXA, anti- coagulative medication, history of arterial or venous thromboem- bolic events, coagulopathy, hematologic disorders, or history of seizures.	(1) intraoperative blood loss, (2) postoperative blood loss (via drain output), (3) postoperative swelling/hematoma formation, (4) visual analog scale (VAS) score, and (5) postoperative opioid use.	Our study found that TXA significantly reduced postoperative blood loss, painful postoperative swelling, and hematoma formation and subsequently reduced postoperative pain and opioid use following the Latarjet procedure.
Kim et al. 2017	Participants underwent primary TSA or primary reverse total shoulder arthroplasty.	RTSAs for cuff tear arthropathy were selected to remove confounders, and, thus, revision and tumor reconstruction cases, sequelae after septic arthritis, and fracture sequelae	Peri-operative blood loss via an intra-articular drain.	The use of a single intravenous dose of TXA immediately prior to RTSA reduces hematologic deterioration postoperatively and the amount of Hemovac drainage. TXA could avoid unnecessary transfusion and its associated medical side effects and cost,
Pauzenberger et al. 2017	All patients scheduled to undergo either primary TSA or reverse total shoulder arthroplasty.	refusal to participate in the study, revision surgery, indication for hemiarthroplasty, known allergy to TXA, anticoagulative medication, severe comorbidities, history of arterial or venous thromboembolic	Peri-operative blood loss via an intra-articular drain	Intravenous administration of TXA successfully reduced mean peri-operative blood drainage, total estimated blood loss, pain during the first post-operative days, and haematoma formation in total shoulder arthroplasty.

		events, coagulopathy, haematological disorders, retinopathy, refusal to receive blood transfusion, pregnancy, or breastfeeding.		
Vara et al. 2018	Patients undergoing primary reverse total shoulder arthroplasty.	minors, acute proximal humeral fracture, concomitant procedures (eg, latissimus dorsi tendon transfer), known allergy to TXA, preoperative anemia (Hb <11 g/dL in women, Hb <12 g/dL in men), refusal of blood products, coagulopathy (thrombophilia, platelet count <150,000 mm3, international normalized ratio >1.4, partial thromboplastin time >1.4 times normal), history of thromboembolic event, major comorbidities (severe pulmonary disease, coronary artery disease, previous myocardial infarction, renal failure), or refusal to give written consent	Intraoperative blood loss.	Patients undergoing primary RTSA, TXA was effective in reducing total drain output, total Hb loss, and total blood loss compared with a placebo control.

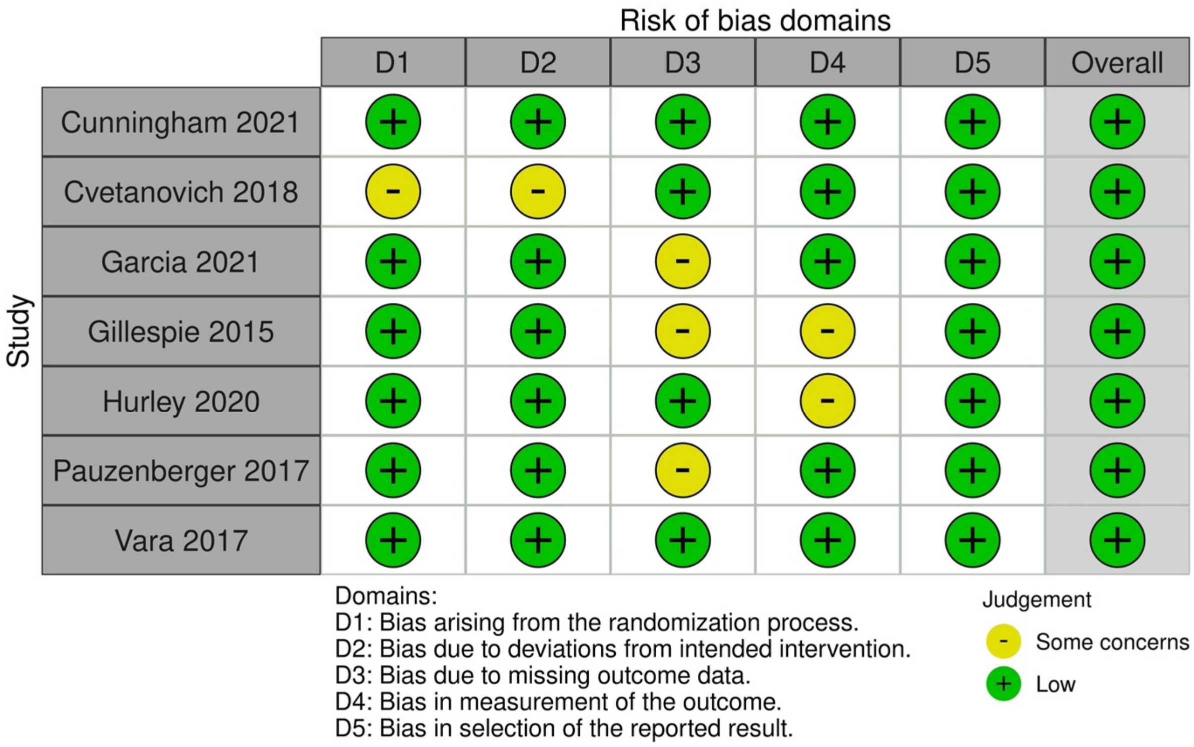

Figure S1. A summary table of review authors' judgements for each risk of bias item for each randomized study.

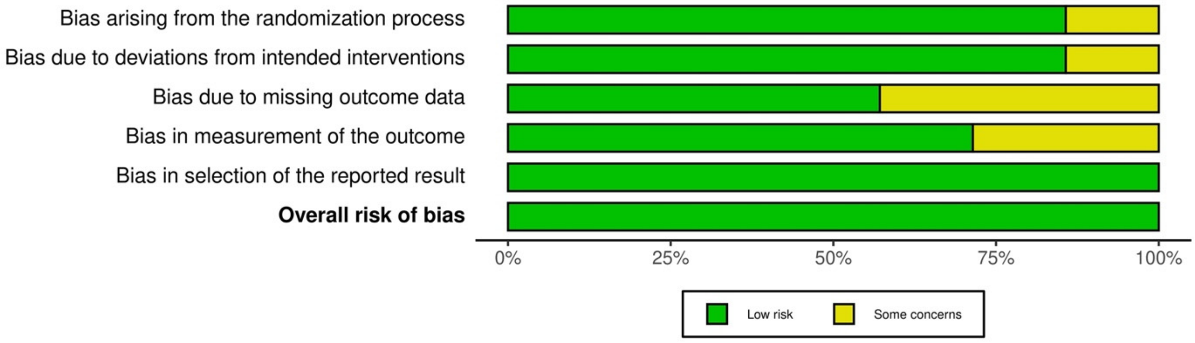

Figure S2. A plot of the distribution of review authors' judgements across randomized studies for each risk of bias item.

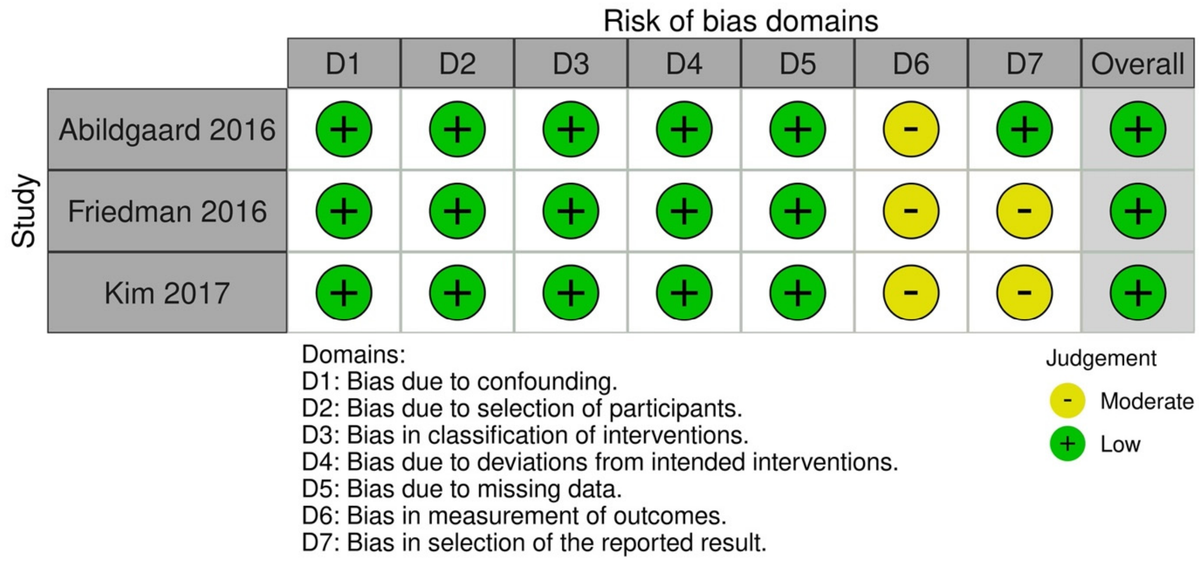

Figure S3. A summary table of review authors' judgements for each risk of bias item for each non-randomized study.

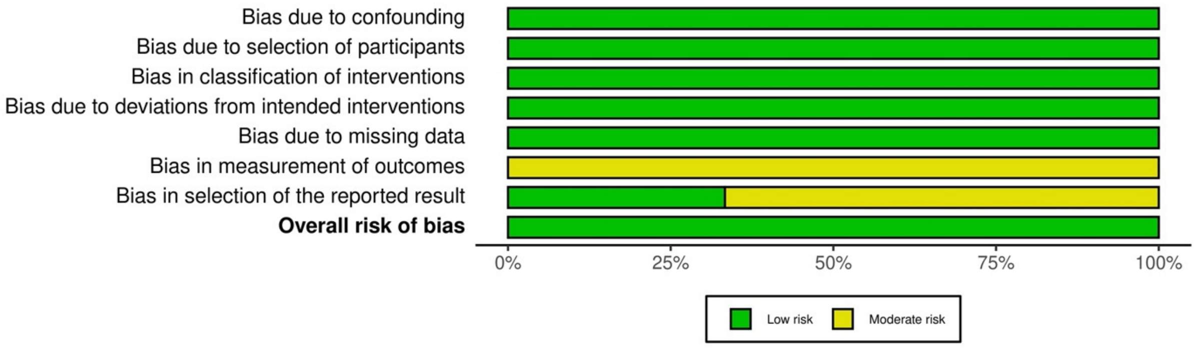

Figure S4. A plot of the distribution of review authors' judgements across non-randomized studies for each risk of bias item.
